# Image reconstruction in non-reciprocal broken-ray tomography

**DOI:** 10.1364/JOSAA.461150

**Published:** 2022-08-18

**Authors:** Matthew J. Faulkner, John C. Schotland, Vadim A. Markel, Lucia Florescu

**Affiliations:** 1 Centre for Vision, Speech and Signal Processing, University of Surrey, GU2 7XH, UK; 2 Department of Mathematics and Department of Physics, Yale University, New Haven, Connecticut 06511, USA; 3 Department of Radiology, University of Pennsylvania, Philadelphia, Pennsylvania 19104, USA

## Abstract

Optical methods of biomedical tomographic imaging are of considerable interest due to their non-invasive nature and sensitivity to physiologically important markers. Similarly to other imaging modalities, optical methods can be enhanced by utilizing extrinsic contrast agents. Typically, these are fluorescent molecules, which can aggregate in regions of interest due to various mechanisms. In the current approaches to imaging, the intrinsic (related to the tissue) and extrinsic (related to the contrast agent) optical parameters are determined separately. This can result in errors, in particular, due to using simplified heuristic models for the spectral dependence of the optical parameters. Recently, we have developed the theory of non-reciprocal broken-ray tomography (NRBRT) for fluorescence imaging of weakly scattering systems. NRBRT enables simultaneous reconstruction of the fluorophore concentration as well as of the intrinsic optical attenuation coefficient at both the excitation and the emission wavelengths. Importantly, no assumption about the spectral dependence of the tissue optical properties is made in NRBRT. In this study, we perform numerical validation of NRBRT under realistic conditions using the Monte Carlo method to generate forward data. We demonstrate that NRBRT can be used for tomographic imaging of samples of up to four scattering lengths in size. The effects of physical characteristics of the detectors such as the area and the acceptance angle are also investigated.

## 1. INTRODUCTION

Optical tomography utilizes near-infrared light to image the optical properties of biological systems [1,2] in a manner that is directly related to tissue function and structure. The use of fluorescent molecular probes has the potential to increase the sensitivity and specificity of the method [3–5]. Fluorescence optical tomography aims to reconstruct the concentration and lifetime of the fluorescent contrast agents. The conventional approaches to fluorescent tomography require the knowledge of the intrinsic optical parameters at both the excitation and emission wavelengths. The latter can be determined separately, but this typically requires some assumptions about the spectral dependence of the absorption and scattering coefficients. As a result, inaccuracies arise in the reconstructed images due to the use of simplistic models of the tissue as well as due to errors in aligning the images provided by different techniques into a single integrated image.

We have recently shown theoretically that fluorescence imaging can be achieved in weakly scattering systems whose size is of the order of a few transport mean free paths [6]. Systems satisfying this condition include epithelial tissue layers, engineered tissue, and some model organisms [7]. Several techniques have been developed for tomographic imaging in this regime [7–20]. However, the wavelength was assumed to be constant in these studies. Inelastic scattering was addressed in [21], where the inversion formula relied on the known angle-dependence energy in Compton scattering and it was assumed that the attenuation coefficient of the medium depends on the energy linearly at each point. In [6], we introduced a tomographic imaging technique, referred to as non-reciprocal broken ray tomography (NRBRT), which enables simultaneous imaging of both the intrinsic and extrinsic optical parameters of a weakly scattering medium [6]. NRBRT accounts for the change in wavelength upon scattering and does not require any assumptions about the spectral properties of the tissue. In the presence of a fluorescent contrast agent, incident light is absorbed by the contrast agent and re-emitted in a different direction and at a different wavelength. In this setting, NRBRT makes use of angularly selective measurements of the fluorescence light exiting the medium at a fixed angle relative to the incident direction. Here the path of light as it travels from the source to the detector is assumed to be a broken ray where the wavelength is changed at its vertex. Image reconstruction in NRBRT involves inversion of the broken-ray transform (BRT)—a generalization of the Radon transform to the case of broken rays, relating measurements to line integrals along broken rays. As a result of the change in the wavelength that occurs at the vertex of the broken ray, different measurements are obtained when the source and detector positions are interchanged. In other words, the resulting BRT is non-reciprocal, meaning that the measurements depend on the direction in which the broken ray is traversed. We have shown that, by exploiting the non-reciprocity of the BRT and by using a measurement geometry with three coplanar broken rays with a common vertex, it is possible to derive inversion formulas for simultaneous reconstruction of the intrinsic attenuation coefficient of the medium at both the excitation and the fluorescence emission wavelengths, as well as the concentration of the fluorophore.

The inverse problem of NRBRT is two-dimensional, since any broken ray can be confined to a plane. Thus, three-dimensional image reconstruction can be performed slice by slice. Moreover, inversion formulas for the BRT can be applied to both backscattering and transmission measurement geometries. Importantly, we note that NRBRT does not require rotating the imaging device around the sample and does not require any assumptions about the spectral dependence of the attenuation coefficient.

In this paper, we demonstrate image reconstruction in NRBRT by performing numerical experiments. For this purpose, forward data are generated by Monte Carlo simulations in inhomogeneous samples characterized by varying scattering strengths and optical parameters for both transmission and backscattering measurement geometries. Although the single-scattering approximation is employed in deriving the reconstruction formula of the NRBRT, in the simulations reported below we do not assume that only singly scattered light is measured by the detectors. Therefore, our simulations are designed to be representative of a potential practical implementation of NRBRT in which all fluorescence light within the geometrical and collimation constraints of a detector is measured.

The rest of this paper is organized as follows. In Sections 2 and 3, we review the principles of NRBRT and present the derivation of the forward data function for the case of finite-size detectors, as well as the image reconstruction formulas used in this study. In Section 4, we describe the simulation of intensity measurements using the Monte Carlo technique. Image reconstructions are presented in Section 5, and Section 6 contains a discussion and conclusions.

## 2. PRINCIPLES OF NRBRT

**Fig. 1.**
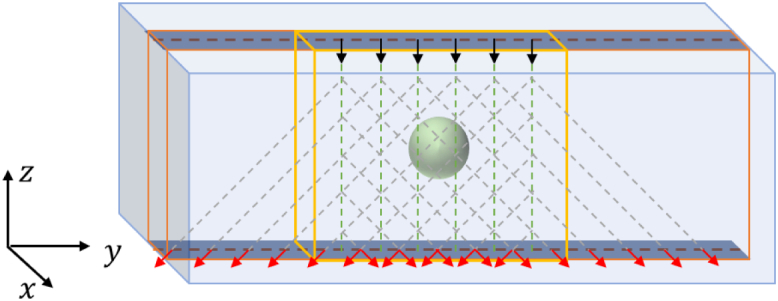
Numerical sample and scanning of a region of interest in a slice with multiple sources and detectors in the transmission measurement geometry. The sphere represents the inhomogeneity and the small cuboid the region of interest (slice) in which the reconstruction is performed. The arrows pointing into the sample represent the incident photons, and those pointing out of the sample the detected fluorescence photons.

**Fig. 2.**
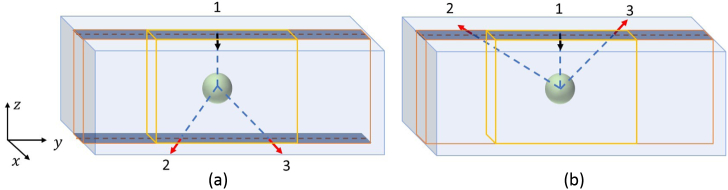
Two measurement geometries for a set of one source and two detectors. The three broken rays corresponding to each of the source-detector and detector-detector pairs are presented by shaded lines. The numbers 1, 2, 3 label the three fixed directions used in this study.

In what follows, we review the principles of NRBRT, which have been introduced in [6]. We consider the problem of fluorescence tomography of samples characterized by spectrally and spatially dependent intrinsic absorption and scattering coefficients, $\mu_{\text{a}}$ and $\mu_{\text{s}}$ , and the spatially dependent fluorophore concentration n . The presence of the fluorophore results in additional absorption, so that the total attenuation coefficient of the sample is $\mu =\mu_{\text{a}}+\mu_{\text{s}}+n\sigma$ , where σ is the spectrally dependent fluorophore absorption cross section. NRBRT reconstructs slice by slice the total attenuation coefficient at the excitation and fluorescence wavelengths, $\mu_{\text{e}}$ and $\mu_{\text{f}}$ , and the fluorophore concentration n . It is based on angularly selective measurements of the fluorescence light intensity on the surface of the sample involving sets of three coplanar broken rays for a given slice of the medium. To implement this, three distinct, fixed directions are used for illumination and detection. As illustrated in Fig. 1, the slice under consideration is illuminated in one of the three directions by collimated, monochromatic sources at a wavelength within the fluorophore resonant absorption band, and the fluorescence photons emerging from the slice in the other two directions are registered. The source and detector positions are chosen so that, for each source, there are sets of three broken rays, corresponding to the source-detector and detector-detector pairs, which lie in the selected slice and have a common vertex. An example of such set of three broken rays is illustrated in Fig. 2. Moreover, for a given region of interest, the number of sources and detectors are such that the region of interest is fully scanned, i.e., all pixels of the discretized grid of the region of interest contain the vertices of three broken rays corresponding to a source and detectors at the two detection angles.

**Fig. 3.**
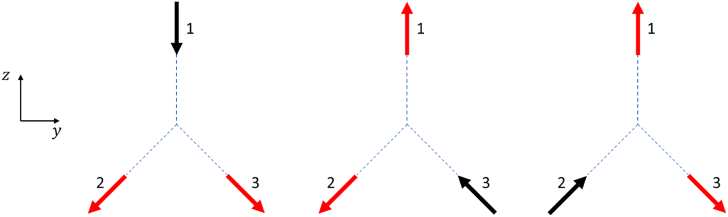
Three measurement configurations for the transmission scan for a set of one source and two detectors. In this study, directions 2 and 3 are mutually orthogonal and are at 135º relative to 1.

Two measurement geometries can be implemented as is illustrated in Fig. 2. To generate a complete data set, scans are performed for a given configuration of source and detector positions and directions, and then the sources and detectors are interchanged, and the sample is scanned again, in each case using the same positions and directions. This results in three scanning configurations for each measurement geometry as is shown in Fig. 3. For each measurement geometry, all the scanning configurations where the sources and detectors are interchanged, and the positions of the sources and detectors varied, represent a complete scan. We note that one of the complete scans consists of both transmission and backscattering measurements. We refer to this as the transmission scan. The other complete scan consists of only backscattering measurements, and we refer to this as the backscattering scan.

## 3. THEORY

### A. Forward Data Function

In [6], the forward data function was derived for the case of infinitely narrow incident beams and infinitely small detectors. In this study, we consider finite-size and finite-collection angle detectors. The signal measured by an angularity selective detector at ${\text{r}}_{\text{d}}$ collimated in the direction ${\hat{\text{s}}}_{k}$ and having an acceptance angle β , for a source at ${\text{r}}_{\text{s}}$ and in the direction ${\hat{\text{s}}}_{l}$ , can be expressed as

(1) $$\delta W_{\text{lk}}({\text{r}}_{\text{s}},{\text{r}}_{\text{d}})=\int_{\delta \Sigma }d^{2}\text{r}\int d^{2}\hat{\text{s}}\eta ({\hat{\text{s}}}_{k}\cdot \hat{\text{s}})({\hat{\text{s}}}_{k}\cdot \hat{\text{s}})I(\text{r},\hat{\text{s}}),$$

where δΣ is the detector area and $\eta ({\hat{\text{s}}}_{k}\cdot \hat{\text{s}})=\eta_{0}\Theta (\beta -\cos^{-1}(\hat{\text{s}}\cdot {\hat{\text{s}}}_{k}))$ is the detector sensitivity, with $\eta_{0}>0$ and Θ(⋅) the unit step function. $I(\text{r},\hat{\text{s}})$ is the specific intensity at the fluorescence wavelength measured at point r and in the direction $\hat{\text{s}}$ and has the expression [6]

(2) $$\begin{array}{ll} & I(\text{r},\hat{\text{s}})=\frac{W\tilde{n}(\text{R})\Theta (\pi -(\theta +\theta_{l}))\delta (\varphi -\pi )}{4\pi \sqrt{\sigma }|\text{r}-{\text{r}}_{\text{s}}|\sin\theta_{l}\sin\theta_{k}}\\ & \;\times \exp\left(-\int_{0}^{L_{1}}\mu_{\text{e}}({\text{r}}_{\text{s}}+\xi {\hat{\text{s}}}_{l})d\xi -\int_{0}^{L_{2}}\mu_{\text{f}}(\text{R}+\xi \hat{\text{s}})d\xi \right). \end{array}$$

**Fig. 4.**
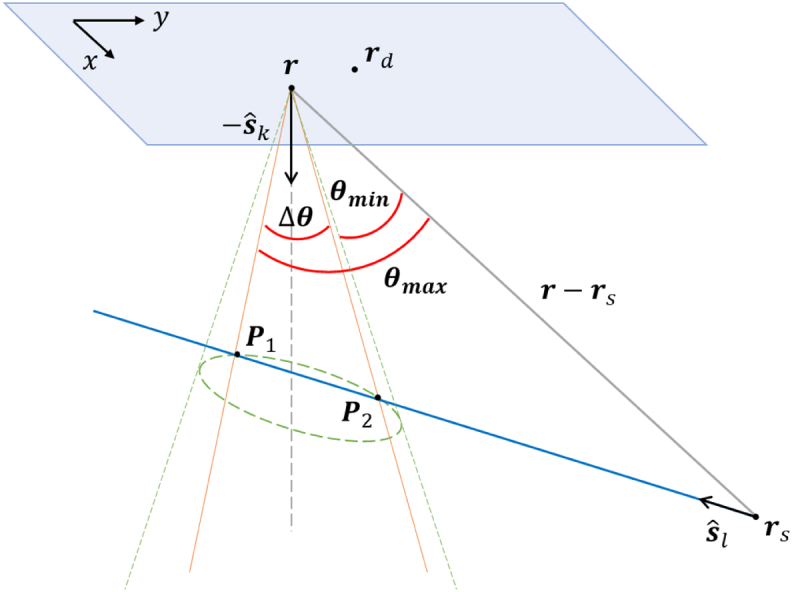
Illustrating the geometry relevant to Eq. (3).

Here W is the source power per unit area; $\text{R}=\text{r}-[|\text{r}-{\text{r}}_{\text{s}}|\sin\theta_{l}/\sin(\theta_{l}+\theta )]\hat{\text{s}}$ is the position of the vertex of the broken ray defined by the source and detector pair; $\tilde{n}(\text{R})=\sigma^{3/2}n(\text{R})$ ; $\theta_{l}$ , $\varphi_{l}$ and θ , φ are the polar angles of the unit vectors ${\hat{\text{s}}}_{l}$ and $\hat{\text{s}}$ , respectively, defined in a reference frame whose z axis coincides with the line of sight $\text{r}-{\text{r}}_{\text{s}}$ , and δ(⋅) is the Dirac delta function. $\mu_{\text{e}}$ and $\mu_{\text{f}}$ are the total attenuation coefficients at the excitation and fluorescence wavelengths, and $L_{1}=|\text{R}-{\text{r}}_{\text{s}}|$ and $L_{2}=|\text{r}-\text{R}|$ represent the lengths of the two rays making up the broken ray defined by the source-detector pair. Note that Eq. (2) was derived under the assumption that ${\hat{\text{s}}}_{l}$ and $\text{r}-{\text{r}}_{\text{s}}$ lie in the same plane [6]. The presence of the delta function and step function ensure that $\hat{\text{s}}$ lies in the same plane as well, and that the rays in the directions $\hat{\text{s}}$ and ${\hat{\text{s}}}_{l}$ intersect in that plane. Using Eqs. (1) and (2), we obtain

(3) $$\begin{array}{ll} & \delta W_{\text{lk}}({\text{r}}_{\text{s}},{\text{r}}_{\text{d}})≃\frac{\eta_{0}W}{4\pi \sqrt{\sigma }}\int_{\delta \Sigma^{\prime }}d^{2}\text{r}\frac{1}{|\text{r}-{\text{r}}_{\text{s}}|\sin\theta_{l}}\\ & \;\times \int_{0}^{\pi }\tilde{n}({\text{R}}^{\prime })\exp\left(-\int_{0}^{L_{1}^{\prime }}\mu_{\text{e}}d\xi -\int_{0}^{L_{2}^{\prime }}\mu_{\text{f}}d\xi \right)\\ & \;\times \Theta (\pi -(\theta +\theta_{l}))\Theta (\beta -\cos^{-1}(\hat{\text{s}}\cdot {\hat{\text{s}}}_{k}))d\theta , \end{array}$$

where we have used that $d^{2}\hat{\text{s}}=\sin\theta d\theta d\varphi$ and made the approximation ${\hat{\text{s}}}_{k}\cdot \hat{\text{s}}≃1$ . Here ${\text{R}}^{\prime }={\text{R}}^{\prime }(\text{r},\theta )$ is the vertex position defined by the source position and direction ${\text{r}}_{\text{s}}$ , ${\hat{\text{s}}}_{l}$ and the point and direction of observation r , $\hat{\text{s}}$ (the latter is in turn defined by θ ), and $L_{1,2}^{\prime }(\text{r},\theta )$ are defined as above but using ${\text{R}}^{\prime }$ instead of R . Note that, as follows from the presence of the step function and is illustrated in Fig. 4, the range of values of θ that yield a nonzero result is restricted to $\left[\theta_{\text{min}},\theta_{\text{max}}\right]$ , where $\theta_{\text{min},\;\text{max}}$ correspond to the intersection of the source ray in the direction ${\hat{\text{s}}}_{l}$ with the acceptance cone of the detector at r collimated in the direction ${\hat{\text{s}}}_{k}$ .

**Fig. 5.**
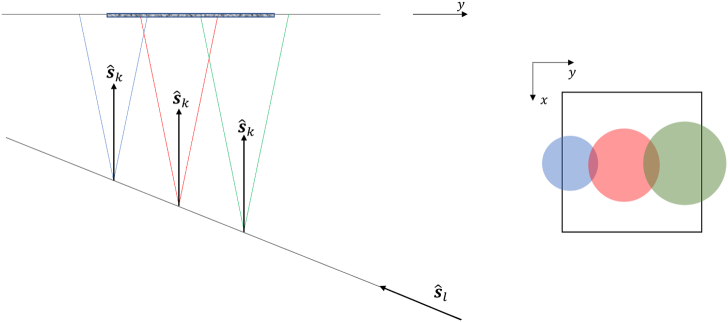
Illustrating detection along the direction ${\hat{\text{s}}}_{k}$ with an acceptance angle β of photons emitted by fluorescence sources along the line defined by the incident source direction ${\hat{\text{s}}}_{l}$ . For a small distance between the fluorescence source and the detector, each fluorescence source contributes to the detected signal with photons detected over areas smaller than the detector area, and not all the extent of the detector area in the x direction is crossed by photons.

**Fig. 6.**
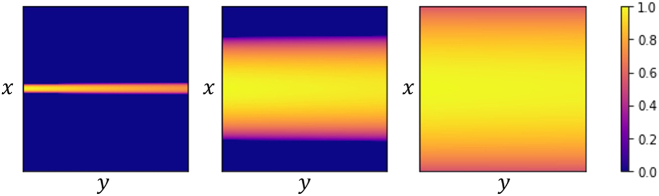
Geometrical factor $f_{31}$ as a function of the position (x,y) on the detector for the transmission measurement. The source line passes through the center of the sample, and the source-detector separation on y direction is, from left to right, 4.85 cm, 2.55 mm, and 0.25 mm. The values have been normalized in each case, and the other parameters are $L_{z}=5.1\;mm$ , β=0.5deg , $\Delta_{\text{d}}=0.07\;mm$ .

For small detector acceptance angles, we can approximate ${\text{R}}^{\prime }(\text{r},\theta )≃{\text{R}}^{\prime }(\text{r},\theta_{0})$ , where $\theta_{0}$ is the angle between ${\hat{\text{s}}}_{k}$ and $\text{r}-{\text{r}}_{\text{s}}$ . This approximation assumes that the sample parameters are constant in the domain within the sample probed by the signal measured at r (the intersection between the cone at r and the source ray). This is a good approximation apart from areas of large variations of the optical parameters such as an inhomogeneity boundary. By using this approximation, we obtain

(4) $$\begin{array}{ll} \delta W_{\text{lk}}({\text{r}}_{\text{s}},{\text{r}}_{\text{d}}) & ≃\frac{\eta_{0}W}{4\pi \sqrt{\sigma }}\int_{\delta \Sigma^{\prime }}d^{2}\text{r}\frac{\Delta \theta_{\text{lk}}(\text{r})}{|\text{r}-{\text{r}}_{\text{s}}|\sin\theta_{l}}\tilde{n}({\text{R}}^{\prime })\\ & \;\times \exp\left(-\int_{0}^{L_{1}^{\prime }}\mu_{\text{e}}d\xi -\int_{0}^{L_{2}^{\prime }}\mu_{\text{f}}d\xi \right), \end{array}$$

where $\Delta \theta_{\text{lk}}(\text{r})=\theta_{\text{max}}-\theta_{\text{min}}$ , and its calculation is presented in Appendix A. The geometrical factor $f_{\text{lk}}(\text{r})=\Delta \theta_{\text{lk}}(\text{r})/|\text{r}-{\text{r}}_{\text{s}}|\sin\theta_{l}$ describes the photon distribution on the detector and is particularly important in the case of large separation (in the y direction) between the source and detector, or, equivalently, for vertex positions and reconstruction points close to the sample surface. In this case, the detector dimension is larger than the other relevant lengths, and not all points on the detector surface are crossed by photons as illustrated in Fig. 5. The factor $f_{\text{lk}}(\text{r})$ accounts for this, as well as for the non-uniform distribution of photons over the detector area. Figure 6 illustrates the factors $f_{\text{lk}}(\text{r})$ for three source-detector configurations that differ only by the detector position.

Further, ${\text{R}}^{\prime }$ is approximated to be the same as the vertex position R defined by the source position and direction ${\text{r}}_{\text{s}}$ , ${\hat{\text{s}}}_{l}$ and the center and collimation direction of the detector ${\text{r}}_{\text{d}}$ , ${\hat{\text{s}}}_{k}$ . Also, we approximate the path of the fluorescence photons from the vertex position to any point r on the detector surface with the path from the vertex position to the center of the detector. This is a good approximation apart from areas of large variation in the sample parameters and situations where the vertex position is close to the surface of the sample where the detector is positioned. With these approximations, we obtain

(5) $$\delta W_{\text{lk}}({\text{r}}_{\text{s}},{\text{r}}_{\text{d}})=\frac{\eta_{0}W}{2\pi \sqrt{\sigma }}{\bar{f}}_{\text{lk}}\tilde{n}(\text{R})\exp\left(-\int_{0}^{{\bar{L}}_{1}}\mu_{\text{e}}d\xi -\int_{0}^{{\bar{L}}_{2}}\mu_{\text{f}}d\xi \right).$$

Here ${\bar{L}}_{1,2}$ are the lengths of the two rays making up the broken ray defined by ${\text{r}}_{\text{s}}$ , ${\hat{\text{s}}}_{l}$ and ${\text{r}}_{\text{d}}$ , ${\hat{\text{s}}}_{k}$ , and

(6) $${\bar{f}}_{\text{lk}}=\int_{\delta \Sigma^{\prime }}\frac{\Delta \theta_{\text{lk}}(\text{r})}{|\text{r}-{\text{r}}_{\text{s}}|\sin\theta_{l}}d^{2}\text{r}$$

represents the counterpart of the geometrical factor for point detectors derived in [6].

Based on Eq. (5), we define the projection data function corresponding to a source at position ${\text{r}}_{\text{s}}$ and in direction ${\hat{\text{s}}}_{l}$ and a finite-size detector at position ${\text{r}}_{\text{d}}$ and in direction ${\hat{\text{s}}}_{k}$ as

(7) $$\phi_{\text{lk}}(\text{R})=-\ln\left[\frac{\delta W_{\text{lk}}}{\eta_{0}W}\frac{2\pi \sqrt{\sigma }}{{\bar{f}}_{\text{lk}}}\right],\;\;\;l,k=1,2,3,\;\;\;l\neq k.$$

We note that, with the data function defined in this way, the forward model has the same form as the forward model for the case of point detectors [6]. Therefore, the image reconstruction formalism derived in [6] can be directly applied here by using the data function defined by Eq. (7).

### B. Image Reconstruction

The sample attenuation coefficient at the excitation and fluorescence wavelengths $\mu_{\text{e}}$ and $\mu_{\text{f}}$ are reconstructed using the image reconstruction formulas derived in [6] for the spectrally averaged attenuation coefficient $\mu^{\left(+\right)}=(\mu_{\text{e}}+\mu_{\text{f}})/2$ , the spectral difference of the attenuation coefficients $\mu^{\left(-\right)}=(\mu_{\text{e}}-\mu_{\text{f}})$ , and $\tilde{n}$ . The reconstruction formulas for $\mu^{\left(\pm \right)}$ are

(8) $$\mu^{\left(\pm \right)}(\text{R})=\mp \frac{1}{\sigma_{1}+\sigma_{2}+\sigma_{3}}\nabla \cdot \Phi^{\left(\pm \right)}(\text{R}).$$

Here $\sigma_{1,2,3}$ are constants determined based on the three source and detector directions, and $\Phi^{\left(\pm \right)}$ are linear combinations of the symmetric and anti-symmetric data functions $\phi_{\text{lk}}^{\left(\pm \right)}$ , with the coefficients of the linear combinations being two-dimensional vectors in the slice plane and depending on the source and detection directions. The details for computing the functions $\Phi^{\left(\pm \right)}$ and the constants $\sigma_{1,2,3}$ are presented in [6]. The symmetric and anti-symmetric data functions $\phi_{\text{lk}}^{\left(\pm \right)}$ are defined as the average and difference of the two datasets measured for a source-detector configuration and for the configuration where the sources and detectors are interchanged, $\phi_{\text{lk}}^{\left(+\right)}=(\phi_{\text{lk}}+\phi_{\text{kl}})/2$ , $\phi_{\text{lk}}^{\left(-\right)}=\phi_{\text{lk}}-\phi_{\text{kl}}$ . The data functions $\phi_{\text{lk}}$ are given by Eq. (7) with the measured signal $\delta W_{\text{lk}}$ computed according to Eq. (14). Once $\mu^{\left(\pm \right)}$ are determined, the attenuation coefficient at the excitation and fluorescence wavelengths are obtained as $\mu_{\text{e,f}}(\text{R})=\mu^{\left(+\right)}(\text{R})\pm (1/2)\mu^{\left(-\right)}(\text{R})$ .

We note that the reconstructions formulas in Eq. (8) are local, which enables reconstructing the attenuation coefficients in the domain where fluorescence contrast agent is present [6]. Also, due the differentiation in Eq. (8), the image reconstruction of $\mu^{\left(\pm \right)}$ and $\mu_{\text{e,f}}$ does not depend on the constants in Eq. (7) for the data function and in the expression of measured signal (presented in Section 4), and therefore the image reconstruction is quantitative.

The reconstruction formula for the concentration of the fluorescent contrast agent reads [6]

(9) $$\tilde{n}(\text{R})=\exp\{I_{l}^{\left(+\right)}(\text{R})+I_{k}^{\left(+\right)}(\text{R})-\phi_{\text{lk}}^{\left(+\right)}(\text{R})\},$$

for a source-detector pair lk , where l and k are two of the three directions in Fig. 2. Here

(10) $$I_{l,k}^{\left(+\right)}(\text{R})=\int_{0}^{\infty }\mu^{\left(+\right)}(\text{R}+\xi {\hat{\text{u}}}_{l,k})d\xi \;,$$

where ${\hat{\text{u}}}_{l,k}$ are unit vectors from the vertex position of the broken ray corresponding to the source-detector pair to the source and detector position, respectively, and $\mu^{\left(+\right)}$ is the reconstructed average attenuation coefficient. Thus, $I_{l,k}^{\left(+\right)}(\text{R})$ represent the integrals of $\mu^{\left(+\right)}$ along lines from the vertex position to the source and detector position, respectively. First, we note that calculating these line integrals is only possible if the attenuation coefficients are known (reconstructed) everywhere along these lines. This is not possible if the contrast agent is accumulated just in a subregion of the sample, in which case the attenuation coefficients can be reconstructed only in that subregion. As a result, if the fluorescence contrast agent is not accumulated everywhere in the sample, $\tilde{n}$ cannot be reconstructed anywhere [6]. We also note that, depending on the source or detector positions or directions, such lines may extend outside the reconstruction (fully scanned) domain as it can be seen in Fig. 1. Therefore, calculating the line integrals in Eq. (10) may require knowledge of the average attenuation coefficient $\mu^{\left(+\right)}$ outside of the reconstruction domain. To avoid this, Eq. (9) can be used to obtain

(11) $$\tilde{n}(\text{R})=\exp\{\phi_{12}^{\left(+\right)}(\text{R})-\phi_{32}^{\left(+\right)}(\text{R})+\phi_{13}^{\left(+\right)}(\text{R})-2I_{1}^{\left(+\right)}(\text{R})\}.$$

Here the index 1 corresponds to the direction perpendicular to the sample surface, and 2, 3 to the other two directions as illustrated in Fig. 2. Thus, $I_{1}^{\left(+\right)}(\text{R})$ in Eq. (11) is calculated along a line that is perpendicular to the sample surface and is always contained in the region where $\mu^{\left(+\right)}$ is known. In this study, Eq. (11) was used to reconstruct the fluorophore concentration. Note that this formula involves (through the symmetric data functions $\phi_{\text{lk}}^{\left(+\right)}$ ) pairs of measurements, with sources and detectors in directions l and k , as well as with the sources and detectors interchanged and having the directions k and l , respectively. The reconstruction formulas derived in [6] can also be applied to the case of conventional fluorescence optical tomography. In this case, the attenuation coefficients $\mu_{\text{e, f}}$ of the medium are known, and the problem is to reconstruct just the fluorophore concentration, which can be accomplished by using the formula

(12) $$\tilde{n}(\text{R})=\exp\{I_{\text{e},l}(\text{R})+I_{\text{f},k}(\text{R})-\phi_{\text{lk}}(\text{R})\}\;,$$

where

(13a) $$I_{l,\text{e}}(\text{R})=\int_{0}^{\infty }\mu_{\text{e}}(\text{R}+\xi {\hat{\text{u}}}_{l})d\xi ,$$

(13b) $$I_{k,\text{f}}(\text{R})=\int_{0}^{\infty }\mu_{\text{f}}(\text{R}+\xi {\hat{\text{u}}}_{k})d\xi .$$

Note that in this case only one source-detector measurement configuration lk is needed, with the sources in direction l and detectors in direction k , and without the need to interchange the sources and detectors. Therefore, image reconstructions can be obtained for any of the six lk pairs, and in practice only one such experimental setup would be needed. If measurements are available for a given source-detector configuration and for the configuration where the source and detector are interchanged, Eq. (9) can also be applied to the case of conventional fluorescence optical tomography. This could potentially lead to better reconstructions, as it can be seen in the results section. $I_{l,k}^{\left(+\right)}(\text{R})$ in Eq. (9) are calculated in this case as the line integrals of the known average attenuation coefficient computed according to Eq. (10).

**Table 1.** Sizes of the Samples Used in This Study^*a*^

	Dimensions				Measurement Configuration			
	
Sample	$L_{x}$	$L_{y}$	$L_{z}$	D	Scan	1	2	3
1	6	44	14.1	8, 3	T	141	281	281
2	6	92	14.1	6	B	141	526	281
3	3	34	5.1	1.5	B	51	189	101
4	3	26	3.7	2.3	B	51	150	87
5, 8, 9, 11, 13	6	16	5.1	2	T	51	101	101
6, 10	6	44	14.1	3	T	141	281	281
7	6	34	5.1	2	T, B	51	101 (T), 189 (B)	101
12	3	22	3.1	1.5	B	51	134	81

^*a*^ From left to right: the sample length (in mm) in the x,y,z direction, $L_{x,y,z}$ ; the diameter/length (in mm) of the inhomogeneity(ies) D ; the scan type, where T and B indicate the transmission and backscattering scan, respectively; and the number of source and detector positions for each of the fixed directions 1, 2, 3 presented in Fig. 2.

Note that the reconstructed values of $\tilde{n}$ depend on the constants in the expression in Eq. (7) of the data function through a global multiplication constant, and quantitative reconstruction is not possible without calibration. The images of the reconstructed fluorophore concentration $\tilde{n}$ presented in the results section correspond to the values obtained based on Eqs. (11), (12), or (9) and normalized to the maximum value of the particular image.

## 4. SIMULATIONS

Projection data used for image reconstruction were generated using the Monte Carlo software package Geant4-based Architecture for Medicine-Oriented Simulations (GAMOS) [22,23] and the Tissue Optics plugin [24]. Monte Carlo simulations account in great accuracy for all relevant processes. Specifically, this software package was used to simulate light transport in inhomogeneous optical samples containing fluorescence contrast agents (fluorophore) and the detection of fluorescence light intensity emerging from the sample.

This study utilized three-dimensional samples with the overall shape of cuboids consisting of a homogeneous background and spherical inclusions representing inhomogeneities, as is illustrated in Fig. 1. The sample scattering strength can be quantified by the optical depth, $L_{z}{\bar{\mu }}_{\text{s}}$ , where ${\bar{\mu }}_{\text{s}}$ is the intrinsic scattering coefficient of the background and $L_{z}$ is the sample length in the z direction. Further, we considered isotropic light scattering, and, to improve computational efficiency, the fluorescent molecules were assumed to have the quantum efficiency of one, and fluorophore absorption and emission spectra were modelled as delta functions. The latter is equivalent to monochromatic excitation and narrowly spectrally filtered detection. Also, the absorption of the fluorescence light by the fluorophore and refraction of rays at the sample boundary have been neglected. Numerical experiments have been performed with a variety of samples characterized by various scattering strengths and contrasts between the inhomogeneity and the background. The sizes of the samples used in this study are presented in Table 1, and the optical characteristics in Table 2.

Each sample was discretized, first, by representing it as a stack of slices drawn parallel to the yz plane and of the thickness 0.1 mm. In the numerical examples below, we only show results for the slice containing the center of the inhomogeneity, but reconstructions in other slices can be similarly obtained. Further, each slice was discretised into voxels of the size $0.1\times 0.1\times 0.1\;{mm}^{3}$ . The sources and detectors used for scanning the slice have been placed on a line parallel to the y axis on the surface of the sample. The source and detector positions were evenly separated for all configurations, with the space between these positions of 0.1 mm. One of the three scanning directions was normal to the surface of the sample. The other two directions were at angles $\alpha =\pm 45^{\circ }$ relative to the normal to the surface, for the transmission scan, and at 45º and $-70^{\circ }$ , for the backscattering scan. The scan type and number of source and detector positions for each sample and direction are listed in Table 1. For each source, $10^{11}$ photons were incident onto the medium. This was implemented by performing 1000 simulations with $10^{8}$ incident photons for each source. Each simulation used a different seed for the pseudorandom number stream and a different position in the stream for the value used to initialize the simulation. The output data were calculated as the sum of the outputs of all simulations.

**Table 2.** Characteristics of the Samples Used in the Simulations^*a*^

	Background				Inhomogeneity				
		
Sample	$L_{z}{\bar{\mu }}_{\text{s};\text{e}}$	$L_{z}{\bar{\mu }}_{\text{s};\text{f}}$	$\frac{{\bar{\mu }}_{\text{a};\text{e}}}{{\bar{\mu }}_{\text{s};\text{e}}}$	$\frac{\sigma \bar{n}}{{\bar{\mu }}_{\text{a};\text{e}}}$	$D\mu_{\text{s};\text{e}}$	$D\mu_{\text{s};\text{f}}$	$\frac{\mu_{\text{e}}}{{\bar{\mu }}_{\text{e}}}$	$\frac{\mu_{\text{f}}}{{\bar{\mu }}_{\text{f}}}$	$\frac{n}{\bar{n}}$
1	0.7	0.63	0.43	4.3	0.8, 0.6	0.7, 0.5	2, 4	2, 4	2, 4
2	0.7	0.6	0.14	5	0.6	0.5	2.6	2	3.5
3	0.7	0.6	0.14	5	0.4	0.4	3.2	2	5.1
4	0.7	0.6	0.1	10	1.1	0.9	2.3	2.5	2
5	1.5	1.0	0.1	5	1.8	1.2	3	3	3
6	1.5	1.0	0.1	3.30	1.3	0.9	2.3	2	3.5
7	1.5	1.0	0.1	0.5	1.8	1.2	3.7	3	18
8	1.5	1.0	0.1	0.25	1.8	1.2	3.7	3	36
9	3.0	2.0	0.1	2.5	3.5	2.4	3	3	3
10	3.0	2.5	0.1	1.6	2.6	2.1	2.2	2	3.5
11	3.0	2.0	0.1	0	3.5	2.4	3.7	3	-
12	3.0	2.0	0.1	0	4.4	2.9	3.7	3	-
13	4.0	3.6	0.1	0	4.7	4.2	3.8	3	-

^*a*^ ${\bar{\mu }}_{\text{s};\text{e}}$ and ${\bar{\mu }}_{\text{s};\text{f}}$ are the intrinsic scattering coefficients of the background at the excitation and fluorescence wavelength, respectively; ${\bar{\mu }}_{\text{a};\text{e}}$ is the intrinsic absorption coefficient of the background at the excitation wavelength; and $\bar{n}$ is the contrast agent concentration in the background. ${\bar{\mu }}_{\text{a};\text{f}}/{\bar{\mu }}_{\text{s};\text{f}}$ is the same as ${\bar{\mu }}_{\text{a};\text{e}}/{\bar{\mu }}_{\text{s};\text{e}}$ for each sample, where ${\bar{\mu }}_{\text{a};\text{f}}$ and ${\bar{\mu }}_{\text{s};\text{f}}$ are the intrinsic absorption and scattering coefficients of the background at fluorescence wavelength. $\mu_{\text{s};\text{e}}$ and $\mu_{\text{s};\text{f}}$ are the scattering coefficients of the inhomogeneity at the excitation and fluorescence wavelengths, respectively. $\mu_{\text{e}}$ , $\mu_{\text{f}}$ and ${\bar{\mu }}_{\text{e}}$ , ${\bar{\mu }}_{\text{f}}$ are the total attenuation coefficients of the inhomogeneity and the background at excitation and fluorescence wavelength, respectively. n is the contrast agent concentration in the inhomogeneity. For samples 11, 12, and 13, $\sigma \bar{n}/{\bar{\mu }}_{\text{e;a}}=7.5,7.5,9$ , respectively.

**Fig. 7.**
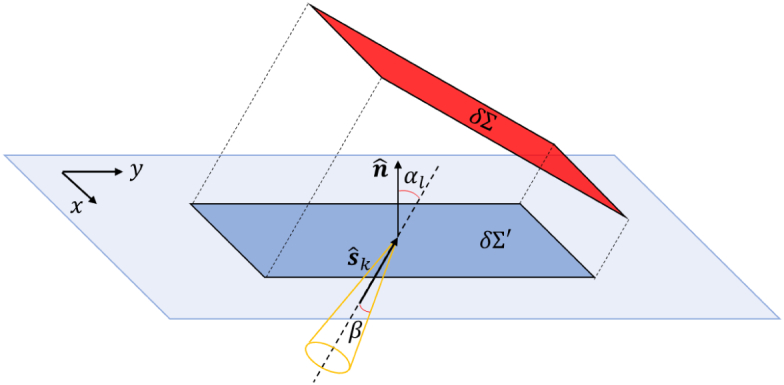
Photon scoring. δΣ represents the collimated detector and $\delta \Sigma^{\prime }$ the projection of the detector onto the sample surface where the photons are scored.

To simulate light-intensity detection by angularly selective detectors represented whose surface is the region δΣ and collimated in the direction defined by the angle α , we have scored the fluorescence photons on the surface of the sample in the region $\delta \Sigma^{\prime }$ representing the projection of the detector onto the sample surface as shown in Fig. 7. Note that there are photons scored close to the edge $\delta \Sigma^{\prime }$ that do not cross δΣ and also photons that cross δΣ that do not cross $\delta \Sigma^{\prime }$ . However, the fraction of $\delta \Sigma^{\prime }$ that scores photons not crossing δΣ , and the fraction of the area δΣ crossed by photons not recorded by $\delta \Sigma^{\prime }$ are of the order of $(1/2)\tan\alpha \sin\beta ≃10^{-2}$ for the typical values of β . For all samples except Samples 2, 3, 4, and 12, we have considered measurements with square detectors of fixed area $\Delta_{\text{d}}^{2}$ . Correspondingly, photons were scored on the sample surface on rectangles (for detection angle α≠0 ) of sides $\Delta_{\text{d}}$ and $\Delta_{\text{d}}/\cos\alpha$ , in the x and y direction, respectively, or squares (for α=0 ). The variable $\Delta_{\text{d}}$ in Section 5 is the side of the squares δΣ . This corresponds to using the same detector for all directions. In contrast, for the backscattering scans of Samples 2, 3, 4, and 12, for all directions, photons were scored on the sample surface on squares of fixed area $\Delta_{\text{d}}^{2}$ , and $\Delta_{\text{d}}$ referred to in Section 5 is the side of these squares. This corresponds to using different detector sizes for different directions. Image reconstruction was better for these samples with such measurements. This is so because, as discussed in Section 6, the data generated by Monte Carlo codes are noisier for backscattering measurements, which affects image reconstruction. The detector configuration that was used for samples 2, 3, 4, and 12 increases the signal-to-noise ratio while keeping the computational time within a reasonable limit.

For a source at position ${\text{r}}_{\text{s}}$ and illuminating in the direction ${\hat{\text{s}}}_{l}$ , we recorded the number $N({\text{r}}_{i},{\hat{\text{s}}}_{j};{\text{r}}_{\text{s}},{\hat{\text{s}}}_{l};\beta )$ of photons (hits) at the fluorescence wavelength emerging from the slice at points ${\text{r}}_{i}$ on the surface and propagating in the direction ${\hat{\text{s}}}_{j}$ within a cone of half-angle β and axis in the direction ${\hat{\text{s}}}_{k}$ and with the apex at ${\text{r}}_{i}$ , where ${\hat{\text{s}}}_{l,k}$ are two of the three distinct directions considered in this study. The mesh for the points ${\text{r}}_{i}$ is fixed in GAMOS and is $10^{-7}\;mm$ . Using Eq. (1), the signal measured by an angularly selective detector positioned at ${\text{r}}_{\text{d}}$ of area δΣ and acceptance angle β and collimated in the direction ${\hat{\text{s}}}_{k}$ was computed as

(14) $$\delta W_{\text{lk}}({\text{r}}_{\text{s}},{\text{r}}_{\text{d}})=\eta_{0}\sum_{{\text{r}}_{i}\in \delta \Sigma^{\prime }}\sum_{j}({\hat{\text{s}}}_{k}\cdot {\hat{\text{s}}}_{j})N({\text{r}}_{i},{\hat{\text{s}}}_{j};{\text{r}}_{\text{s}},{\hat{\text{s}}}_{l};\beta )E_{f},$$

where $\delta \Sigma^{\prime }$ is centered at ${\text{r}}_{\text{d}}$ , and $E_{f}$ is the energy of fluorescence photons. This quantity was then used in Eq. (7) to calculate the data functions used for image reconstruction.

**Table 3.** RMSE and SSIM between the Reconstructed Images and the Corresponding Model for Different Numbers of Incident Photons per Source Position N for Sample 1, Using the Transmission Scan with $\beta =0.5^{\circ }$ , $\Delta_{\text{d}}=0.070\;mm$ and λ=5 for $N=10^{9}$ and $N=10^{10}$ , and λ=20 for $N=5\times 10^{10}$

	$\mu^{\left(+\right)}$		$\mu^{\left(-\right)}$		$\mu_{\text{e}}$		$\mu_{\text{f}}$		n	
					
N	RMSE	SSIM	RMSE	SSIM	RMSE	SSIM	RMSE	SSIM	RMSE	SSIM
$10^{9}$	0.14	0.37	0.11	0.46	0.16	0.39	0.15	0.24	0.25	0.37
$10^{10}$	0.06	0.75	0.05	0.78	0.06	0.75	0.06	0.68	0.17	0.64
$5\times 10^{10}$	0.04	0.86	0.04	0.83	0.05	0.82	0.04	0.84	0.09	0.82

Implementing the reconstruction formulas in Eq. (8) requires numerical differentiation of the functions $\Phi^{\left(\pm \right)}$ . Projection data generated using Monte Carlo simulations are inherently noisy. In addition, due to the presence of sharp-edge inhomogeneities, the data functions are not smooth. Numerical differentiation methods (for example, finite-difference approximations) are known to amplify the noise in the data [21,25,26]. Denoising the data prior to differentiation was found not to always solve the problem [25]. Therefore, a technique based on the regularisation of the derivative [26] was used in this study. This method uses total-variation regularization and is suitable for noisy and discontinuous functions. We used this method following the application of a median filter to $\Phi^{\left(\pm \right)}$ . The total-variation regularization method is based on using periodic boundary conditions for Fourier-domain processing, which resulted in artefacts in the reconstruction at the edges of the region of interest [26]. To reduce these artefacts, after applying the median filter, the functions $\Phi^{\left(\pm \right)}$ were extrapolated to relocate the area most affected by the inherent artefacts of the derivative method outside the region of interest. Specifically, 10 pixels at the edge of the region of interest on all sides were duplicated outside the region of interest. Furthermore, to improve the accuracy of the derivative computation (affected by large local variations of the data functions), the regularization method was applied using as base derivative method both the forward and backward finite difference approximations, and the derivatives in Eq. (8) have been calculated using the second-order finite difference approximation. The value of the regularization parameter λ in the formalism can be varied to obtain an optimal reconstructed image quality.

In implementing the reconstruction formulas from Eqs. (11), (12), and (9), a median filter was applied to the all the data functions $\phi_{\text{lk}}$ used and to the reconstructed $\mu^{\left(+\right)}$ , before the line integrals and $\tilde{n}$ have been computed.

## 5. RESULTS

In this section, we present image reconstructions for the various samples described above. For all the figures, the horizontal and vertical axes of the images correspond to the y and z axes in Fig. 3, respectively, and the same color scale is used for the reconstructed quantities and the corresponding models. The color scale for $\mu^{\left(\pm \right)}$ , $\mu_{\text{e}}$ , and $\mu_{\text{f}}$ has been clipped in the range of 0 to 1.5 times the maximum value in the model. The parameters β , $\Delta_{d}$ , and λ in each case have been chosen to obtain optimal quality of the reconstructed images, based on visual inspection. Further, the quality of these reconstructed images is assessed using the root mean square error (RMSE) and the structural similarity index measure (SSIM) [27], measuring the quantitative and perceptual difference, respectively, between the reconstructed image and the model. Small (close to zero) values of RMSE and values of SSIM close to one indicate good agreement. Table 3 presents the reconstruction errors as a function of the incident number of photons per source, for Sample 1. Based on this, we obtain that acceptable reconstruction is possible for $10^{10}$ or more incident photons. The reconstructions presented below correspond to $10^{11}$ photons per source, and the error metrics for these reconstructions are shown in Table 4.

**Table 4.** RMSE and SSIM between the Reconstructed Image and the Corresponding Model for the Reconstructions Presented

(a) in Figs. 8–12											
		$\mu^{\left(+\right)}$		$\mu^{\left(-\right)}$		$\mu_{\text{e}}$		$\mu_{\text{f}}$		n	
						
Fig.	Sample	RMSE	SSIM	RMSE	SSIM	RMSE	SSIM	RMSE	SSIM	RMSE	SSIM
8	1 ( $\Delta_{\text{d}}=0.070\;mm$ , $\beta =0.75^{\circ }$ )	0.03	0.88	0.04	0.86	0.04	0.85	0.03	0.86	0.05	0.91
	1 ( $\Delta_{\text{d}}=0.070\;mm$ , $\beta =0.5^{\circ }$ )	0.04	0.85	0.04	0.84	0.05	0.82	0.04	0.83	0.07	0.87
	1 ( $\Delta_{\text{d}}=0.070\;mm$ , $\beta =0.25^{\circ }$ )	0.07	0.75	0.06	0.72	0.08	0.72	0.07	0.72	0.07	0.78
	1 ( $\Delta_{\text{d}}=0.028\;mm$ , $\beta =0.5^{\circ }$ )	0.1	0.63	0.09	0.6	0.11	0.61	0.1	0.58	0.14	0.67
9	2	0.3	0.45	0.28	0.41	0.34	0.44	0.32	0.39	0.12	0.72
	3	0.18	0.41	0.19	0.35	0.24	0.34	0.16	0.39	0.13	0.75
	4	0.19	0.48	0.16	0.48	0.25	0.45	0.16	0.44	0.21	0.62
10	5	0.11	0.68	0.09	0.63	0.14	0.62	0.09	0.68	0.11	0.73
	6	0.05	0.8	0.05	0.75	0.06	0.76	0.06	0.76	0.11	0.79
	7 (T)	0.16	0.42	0.14	0.38	0.19	0.39	0.16	0.37	0.1	0.87
	7 (B)	0.52	0.3	0.43	0.3	0.62	0.31	0.5	0.24	0.12	0.63
	8	0.18	0.42	0.16	0.34	0.22	0.37	0.16	0.38	0.1	0.86
11	9	0.32	0.46	0.2	0.43	0.4	0.41	0.26	0.45	0.22	0.5
	10	0.17	0.54	0.09	0.51	0.18	0.53	0.17	0.47	0.24	0.49
12	11	1.66	0.05	1.1	0.07	1.88	0.05	1.67	0.05	-	-
	12	2.91	0.05	0.81	0.01	3.13	0.04	2.78	0.05	-	-
	13	1.3	0.04	0.81	0.06	1.34	0.03	1.42	0.04	-	-

(b) in Fig. 13												
	13		31		32		23		12		21	
						
Sample	RMSE	SSIM	RMSE	SSIM	RMSE	SSIM	RMSE	SSIM	RMSE	SSIM	RMSE	SSIM
7	0.16	0.88	0.16	0.87	0.08	0.85	0.08	0.84	0.16	0.88	0.15	0.88
9	0.27	0.49	0.29	0.42	0.4	0.11	0.4	0.11	0.25	0.53	0.3	0.4
11	0.22	0.66	0.35	0.64	0.24	0.5	0.24	0.51	0.22	0.66	0.33	0.66
13	0.26	0.55	0.33	0.67	0.28	0.53	0.29	0.52	0.26	0.54	0.34	0.66

(c) in Fig. 14						
	13 & 31		32 & 23		12 & 21	
			
Sample	RMSE	SSIM	RMSE	SSIM	RMSE	SSIM
7	0.15	0.89	0.07	0.89	0.15	0.9
9	0.23	0.59	0.34	0.23	0.22	0.59
11	0.35	0.63	0.22	0.51	0.34	0.66
13	0.33	0.67	0.34	0.47	0.34	0.66

**Fig. 8.**
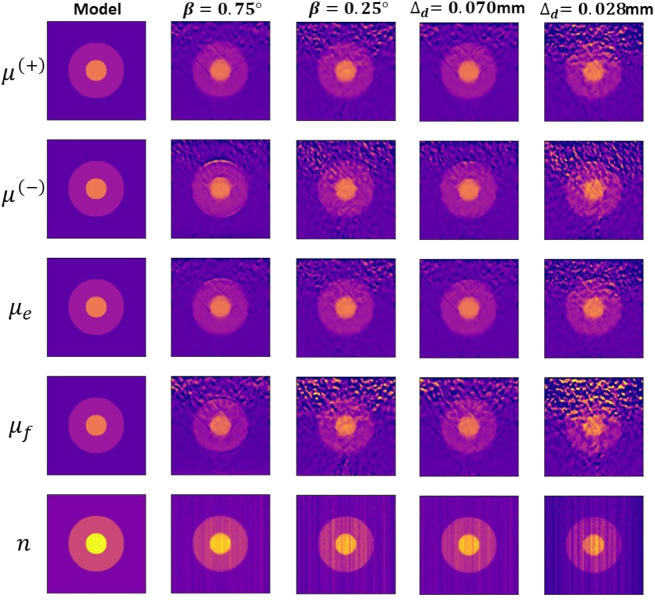
Reconstructed optical parameters of Sample 1 using the transmission scan with $\Delta_{\text{d}}=0.070\;mm$ and $\beta =0.75^{\circ },0.25^{\circ }$ ; $\beta =0.5^{\circ }$ and $\Delta_{\text{d}}=0.070\;mm$ , 0.028mm . For all cases, λ=50 .

**Fig. 9.**
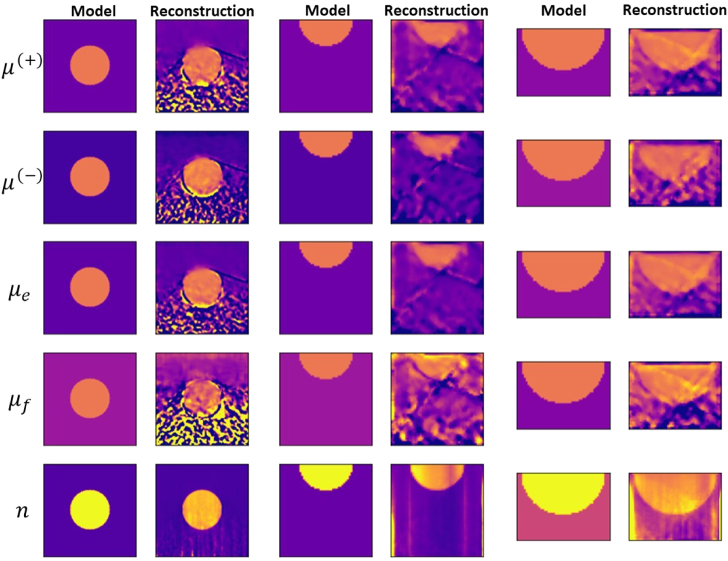
Image reconstruction for Samples 2 (left), 3 (middle), and 4 (right) based on the backscattering scan with $\beta =0.5^{\circ }$ and $\Delta_{\text{d}}=0.1\;mm$ , and for λ=30 , 100, 200, respectively.

The reconstructed optical parameters of Sample 1, characterized by an optical depth of less than 1, based on the transmission scan, are presented in Fig. 8 for varying detector acceptance angles and detector areas. We obtain a good agreement between the reconstructed quantities and the model. The reconstructed image quality is better for larger detector acceptance angles and sizes. Figure 9 presents the reconstructed optical characteristics of Samples 2, 3, and 4, based on the backscattering scan. These samples are all characterized by the same optical depth of 0.7, but they have different sizes and contrasts in optical characteristics between the inhomogeneity and the background as presented in Table 2. We obtain a reasonable agreement between the reconstructed quantities and the model, but the image quality in this case is lower than it is for transmission scans, particularly for regions deeper into the sample and behind the inhomogeneity.

**Fig. 10.**
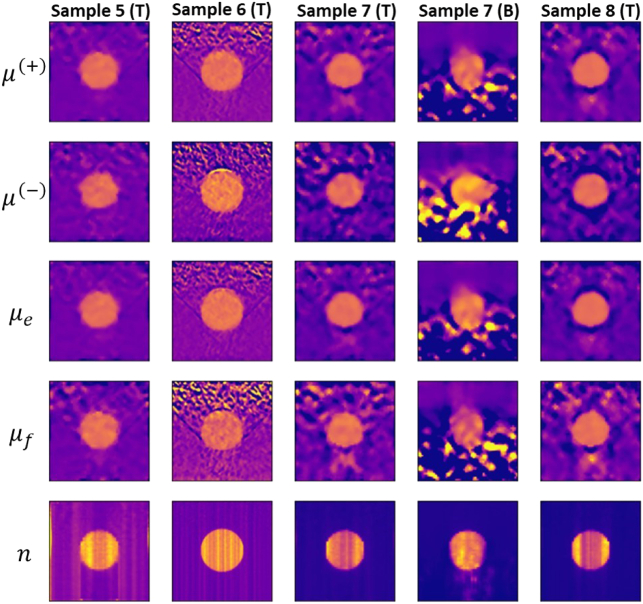
Reconstructed images for (from left to right) Sample 5 using the transmission scan with $\beta =0.25^{\circ }$ , $\Delta_{\text{d}}=0.070\;mm$ , and λ=250 ; Sample 6 using the transmission scan with $\beta =0.5^{\circ }$ , $\Delta_{\text{d}}=0.070\;mm$ , and λ=50 ; Sample 7 using the transmission scan with $\beta =0.25^{\circ }$ , $\Delta_{\text{d}}=0.070\;mm$ , and λ=100 ; Sample 7 using the backscattering scan with $\beta =0.5^{\circ }$ , $\Delta_{\text{d}}=0.034\;mm$ and λ=25 ; and Sample 8 using the transmission scan with $\beta =0.25^{\circ }$ , $\Delta_{\text{d}}=0.070\;mm$ and λ=50 . T and B indicate the transmission and backscattering scan, respectively.

Next, we consider the image reconstruction of stronger scattering samples. Figure 10 presents the reconstructed optical parameters of Samples 5, 6, 7, and 8. All these samples are characterized by the same optical depth of 1.5, but have different sizes, contrast agent concentration in the background, and contrast in optical parameters between the inhomogeneity(ies) and the background. Specifically, Sample 6 is larger and has a larger inhomogeneity than Sample 5, and a lower concentration of the contrast agent in the background; Sample 7 has the same characteristics as Sample 5, apart from a 10 times less contrast agent concentration in the background and a higher contrast between the inhomogeneity and the background for the fluorescence contrast agent; Sample 8 has an even lower concentration of the contrast agent in the background. We obtain good quality images for transmission scans and poorer quality for backscattering scans. Also, the image quality degrades for lower fluorescence contrast agent concentration in the background. However, the inhomogeneity is still visible in all reconstructions.

**Fig. 11.**
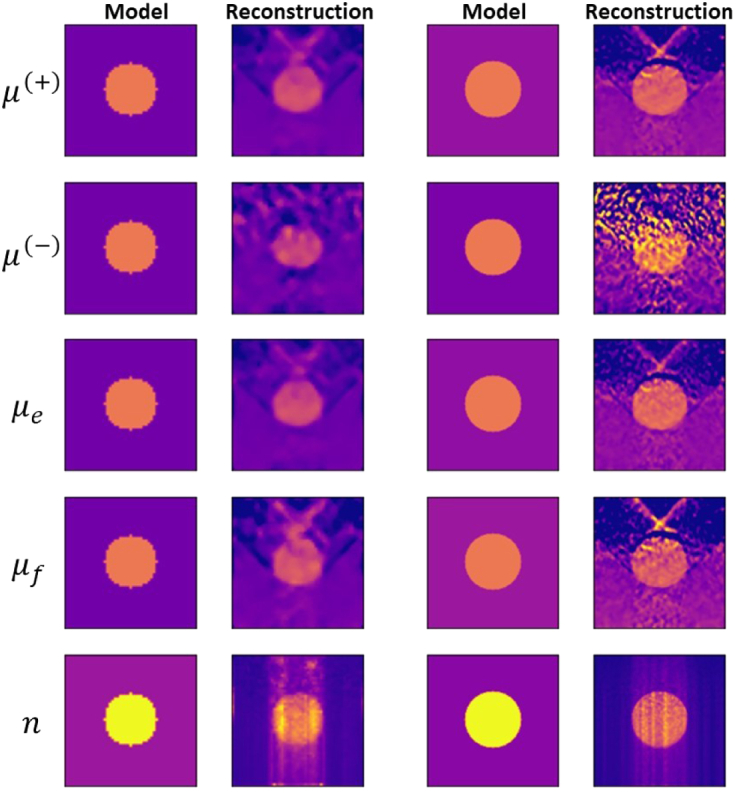
Image reconstruction based on the transmission scan with $\Delta_{\text{d}}=0.070\;mm$ for Sample 9 (left) for $\beta =0.25^{\circ }$ and λ=50 and Sample 10 (right) for $\beta =0.5^{\circ }$ and λ=25 .

The scattering strength was further increased to an optical depth of 3 , for which the single-scattering approximation used in deriving the image reconstruction algorithm is expected to start breaking down. Figure 11 presents the reconstructed optical parameters based on the transmission scan of Samples 9 and 10, differing by their size and contrast agent concentration in the background and by the optical depth of the inhomogeneity. While the quality of the reconstructed images has deteriorated compared to weaker-scattering samples for the same type of scan, the inhomogeneity can still be distinguished with good accuracy, particularly if $\mu^{\left(+\right)}$ , $\mu_{\text{e}}$ , and n are used as markers.

Consider now the case where the contrast agent is exclusively accumulated in the inhomogeneity. In this case, as discussed above, using the reconstruction formalism and the measurements considered in this study, it is possible to reconstruct only the coefficients $\mu^{\left(\pm \right)}$ and $\mu_{\text{e, f}}$ within the domain where the contrast agent is accumulated (the inhomogeneity), but the contrast agent concentration cannot be reconstructed anywhere. RMSE and SSIM for these reconstructed images were computed only for the domain where reconstruction was possible.

**Fig. 12.**
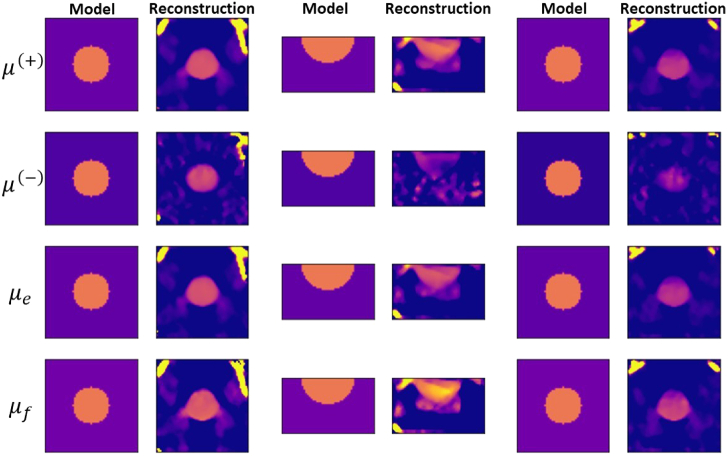
Image reconstruction for Sample 11 (left) using the transmission scan with $\beta =0.25^{\circ }$ , $\Delta_{\text{d}}=0.070\;mm$ , and λ=50 ; Sample 12 (middle) using the backscattering scan with $\beta =0.5^{\circ }$ , $\Delta_{\text{d}}=0.1\;mm$ , and λ=100 ; and Sample 13 (right) using the transmission scan with $\beta =0.25^{\circ }$ , $\Delta_{\text{d}}=0.070\;mm$ , and λ=50 .

Figure 12 presents the reconstructed images for Samples 11 and 12, which have the optical depth of 3, and Sample 13, with an optical depth of 4. The transmission scan was used for Samples 11 and 13 and the backscattering scan for Sample 12. We obtain that the quality of the reconstructed images, and the agreement between the reconstructed images and the model are reasonably good even for these stronger scattering samples.

**Fig. 13.**
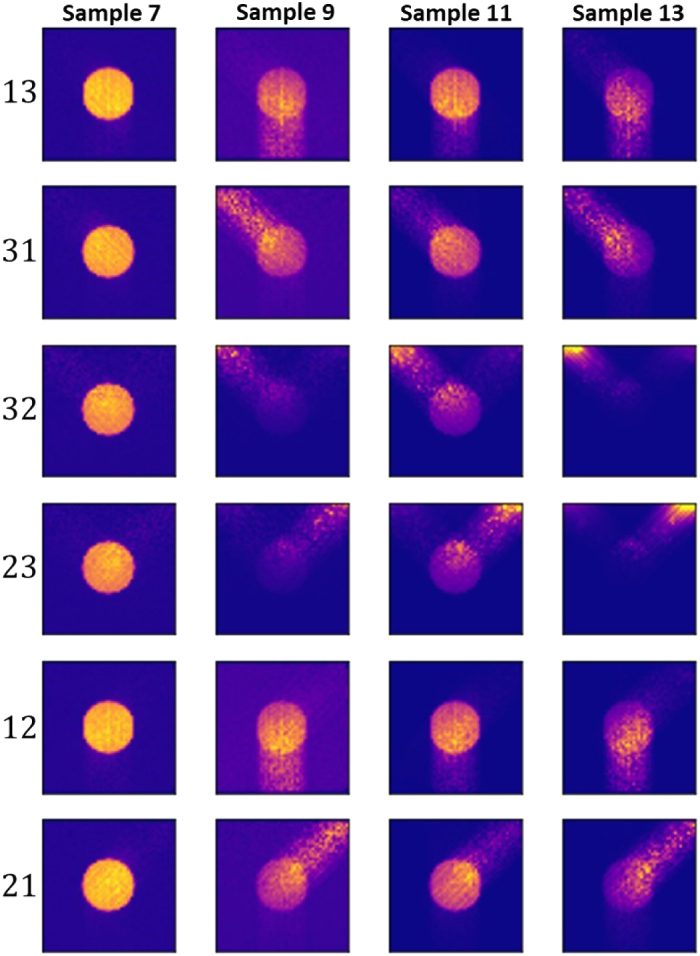
Reconstruction of the fluorescence contrast agent distribution of, from left to right, Samples 7, 9, 11, amd 13, based on the source-detector configurations in Fig. 3, with $\beta =0.25^{\circ }$ and $\Delta_{\text{d}}=0.070\;mm$ , respectively. The labels lk with l,k=1,2,3 on the left indicate the source-detector measurement setup with the source in direction l and detector in direction k .

**Fig. 14.**
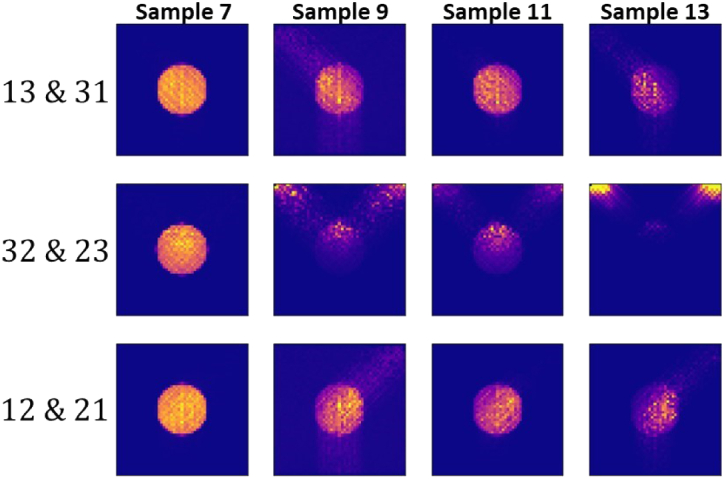
Reconstruction of the fluorescence contrast agent distribution of, from left to right, Samples 7, 9, 11, and 13, based on the pairs of source-detector configurations in Fig. 3 with $\beta =0.25^{\circ }$ and $\Delta_{\text{d}}=0.070\;mm$ , respectively. The labels lk & kl with l,k=1,2,3 on the left indicate that the projection data was the average data corresponding to the source-detector measurement configurations lk and kl .

Finally, we consider the case of conventional fluorescence optical tomography. Figure 13 presents the reconstructed images for Samples 7, 9, 11, and 13 based on Eq. (12) and each of the source-detector measurement configurations that were illustrated in Fig. 3. For samples with optical depth of 1.5 (Sample 7), very good image reconstruction is obtained for all source-detector configurations, with the inhomogeneity distinguished in all reconstructed images. For stronger-scattering samples, good image reconstruction is obtained for transmission measurements. For samples of the optical depth of 3, relatively good reconstruction is possible also for backscattering measurements if the contrast agent is exclusively accumulated in the inhomogeneity. This is also indicated by the SSIM value, which increases by nearly 5 times when comparing Sample 11 to Sample 9. Figure 14 presents the reconstructions for the same samples, but based on Eq. (9) and pairs of measurements, corresponding to a given source-detector configuration and to the configuration where the source and the detector are interchanged. This shows that additional measurements enable reducing the artefacts in the background and better identifying the inhomogeneity.

## 6. DISCUSSION AND CONCLUSIONS

This computational study demonstrated simultaneous reconstruction based on NRBRT of three different quantities characterizing an optical sample, namely, the attenuation coefficient at the excitation and fluorescence wavelengths, $\mu_{\text{e, f}}$ (retrieved via the reconstruction of the average and difference attenuation coefficients $\mu^{\left(\pm \right)}$ ), and the fluorescence contrast agent distribution n . The image reconstruction for $\mu^{\left(+\right)}$ and $\mu_{\text{e}}$ was generally better than for $\mu^{\left(-\right)}$ and $\mu_{\text{f}}$ . This is so because it involves addition of data functions, while the reconstruction of $\mu^{\left(-\right)}$ and $\mu_{\text{f}}$ involves subtraction of (positive) noisy functions, resulting in a reduced signal-to-noise ratio. Similarly, the reconstruction of n was relatively stable. This is so because this reconstruction utilizes the symmetric data function (involving summation) and the relatively stable reconstruction of $\mu_{\text{e}}$ . This study suggests that, for situations wherein the reconstruction of $\mu_{\text{f}}$ is of poor quality, $\mu^{\left(+\right)}$ and $\mu_{\text{e}}$ and n can be used as three independent markers, and therefore comprehensive assessment is still possible. We emphasize that, while the image reconstruction algorithm is based on the single-scattering approximation, the reconstructed images presented above were obtained from simulated data accounting for all orders of scattering.

**Fig. 15.**
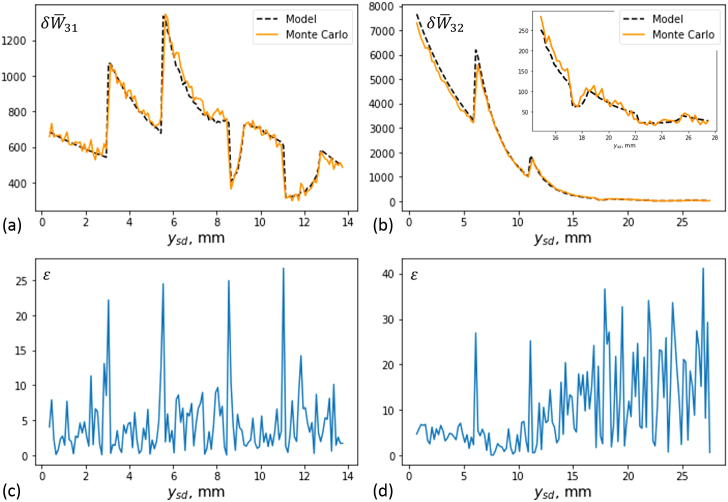
Measured signal, computed based on Monte Carlo simulations and the forward model, and the noise ε in the simulated values as functions of the source-detector separation on the y direction for Sample 1, for (a) and (c) transmission measurements and for (b) and (d) backscattering measurements. The noise was calculated as the percentage relative difference between the simulated signal value and the model signal value. The detector characteristics are $\beta =0.5^{\circ }$ , $\Delta_{\text{d}}=0.070\;mm$ .

Image reconstruction based on the backscattering scan was not as stable as that for the transmission scan. This is so due to the higher levels of noise in the projection data for backscattering measurements. To illustrate this, we considered sets of transmission and backscattering measurements for Sample 1, corresponding to the source-detector pairs 31 and 32 presented in Fig. 3, respectively. The points in the sample probed by each set of measurements (the vertex positions) were the same (and positioned on a line parallel to the z axis and passing through the center of the inhomogeneity). Figure 15 presents the measured signal $\delta {\bar{W}}_{\text{kl}}=\delta W_{\text{kl}}/\eta E_{\text{f}}$ , computed using Monte Carlo simulations and Eq. (14), as a function of the source-detector separation. In this figure, we also present the (scaled) theoretical prediction for the measured signal, calculated using the forward model given in Eq. (5) and the values of $\mu_{\text{e, f}}$ and n of the model. The theoretical signal is determined based on the assumption that no scattering event takes place apart from the inelastic scattering of photons interacting with the contrast agent. This is a good approximation for the weakly scattering Sample 1, and therefore no significant systematic difference between the theoretical and simulated signals is expected. Indeed, it can be seen from the data of Fig. 15 that the theoretical and simulated signals have the same behavior. However, the Monte Carlo signal is noisy, with the noise relatively high for the backscattering measurements, particularly, when probing positions deeper into the sample (which correspond to large source-detector separations). This strongly affects the image reconstruction based on a complete scan consisting only of backscattering measurements, such as the backscattering scan. There is also an effect even for the transmission scan, due to the backscattering measurement components (the set of measurements for the source-detector pairs 23 and 32 presented in Fig. 3), and this explains the artefacts above the inhomogeneity in the reconstructed images presented in this study. While numerical simulations and the levels of noise generated using the available Monte Carlo code and computational resources are not necessarily representative of the experimental situation, indeed, in practice, a lower signal-to-noise ratio (due to both lower signal and higher noise) is expected for the backscattering measurements than for the transmission measurements proposed here. In particular, the lower signal is due in turn to the longer photon paths in the medium, and there could be situations where no signal is measured. In this study, the incomplete dataset was corrected to some extent by using the median filter. Further improvements can be achieved by interpolation or extrapolation, or by appropriately choosing the angles of illumination and detection that determine the photon path length. Also, using sensitive enough detectors and optimal denoising techniques could, in principle, reduce the noise in the measured signal. All these in turn could enable better image reconstructions than in these numerical experiments. Image reconstruction for the transmission scan could also be improved by considering a trade-off for the photons path lengths between transmission and backscattering measurement components, by using different directions of illumination and detection than presented here.

We also note that the measurement geometry with two of the relevant directions at angles $\pm 45^{\circ }$ and the symmetric samples considered in this study for the transmission scan is the most stringent test for image reconstruction since, in this case, the anti-symmetric data functions used in reconstructions are characterized by a relatively small signal-to-noise ratio due to subtraction of two similar noisy quantities. Further improvement in image reconstruction can be achieved by suitably choosing the directions involved to avoid symmetry in the star formed by the three broken rays defined by the illumination and detection directions. In this study, image reconstruction for the backscattering scan was indeed improved by choosing the two directions to be at angles 45º and $-70^{\circ }$ . In practice, insight for the optimization of the directions for a particular sample can be obtained from a preliminary scan and reconstruction based on a chosen set of directions.

Some of the artefacts in the reconstructed images, particularly the streak artefacts, are caused by the sharp discontinuity in the optical characteristics, resulting in sharp variations in the detected signal and data functions as can also be seen in Fig. 15. Although the method used to compute the derivatives involved in the reconstruction formulas is suitable for discontinuous functions [26], its performance has been compromised by the presence of high levels of noise. However, in practice, there is usually a smoother variation in the optical characteristics than the step-like discontinuity considered here. This, together with possibly lower noise levels in the measured signal, would ameliorate the artefacts.

The detector size and acceptance angle both influence the quality of image reconstruction, and a reduction of noise in the measured signal and potentially better reconstruction can be obtained as both these detector characteristics are increased. However, increasing the detector acceptance angle increases the size of the domain within the sample probed by the signal measured at a particular point on the detector (represented by $|{\text{P}}_{1}-{\text{P}}_{2}|$ in Fig. 4), and there could be situations where the optical properties of the sample in that domain are not constant or even exhibit large variations. This possibility was ignored in the derivation of the reconstruction algorithm as discussed in Sec. 3. Therefore, as the detector acceptance angle is increased, the image reconstruction could deteriorate, particularly in areas of variations in the sample parameters. Thus, a trade-off between the measurement noise and the applicability of the forward model needs to be considered when deciding the detector parameters, to obtain the best reconstructions. For this reason, the best reconstructions for some of the samples studied here were obtained for smaller acceptance angles.

Image reconstruction for weakly scattering samples was relatively good. The reconstruction quality deteriorated as the sample optical depth of the background was increased above 4. The breakdown of the reconstruction formalism based on single-scattering approximation is indeed expected as the scattering strength is increased. This is exacerbated by the fact that the detector has a nonzero acceptance angle, and, as a result, the contribution to the detected signal of multiple scattered light could be significant, particularly for stronger scattering samples. In this numerical study, the detector acceptance angle needed to be above a certain value, to maintain a reasonable signal-to-noise ratio for the detected signal while keeping the number of simulations and the computational time within a reasonable limit. However, further reducing the acceptance angle could be possible in practice, potentially extending the applicability of NRBRT to even stronger scattering samples.

In samples with lower fluorescence contrast agent uptake in the background, the inhomogeneity was reconstructed to a good quality, whereas the reconstruction of the background presented artefacts. This was due to low signal-to-noise ratio for measurements probing the background of the sample, resulting from low emitted signal. The ability to reconstruct the inhomogeneity with good accuracy highlights the advantage of an imaging technique with a local reconstruction formula such as the one considered here.

In conclusion, using numerical experiments, this study has validated the principles and image reconstruction formalism of NRBRT applied to fluorescence optical tomography. Detailed guidance was also provided for experimental implementation, as well as possibly a motivation for advancements in areas such as sensitive detection or data denoising while maintaining features and discontinuities and accurate derivative calculation for such data. Future work will include further development and validation of NRBRT to the case of contrast agent exclusively accumulated in the inhomogeneity.

## Data Availability

Data underlying the results presented in this paper are not publicly available at this time but may be obtained from the authors upon reasonable request.

## APPENDIX A: THE INTERSECTION OF THE INCIDENT SOURCE AND THE DETECTOR’S ACCEPTANCE CONE

Consider light incident onto a medium at the position ${\text{r}}_{\text{s}}$ in the direction ${\hat{\text{s}}}_{l}$ and the detector of area $\delta \Sigma^{\prime }$ and acceptance angle β centered at ${\text{r}}_{\text{d}}$ and collimated in the direction ${\hat{\text{s}}}_{k}$ . An arbitrary point X on the incident ray at a distance ξ from ${\text{r}}_{\text{s}}$ is defined by $\text{X}={\text{r}}_{\text{s}}+\xi {\hat{\text{s}}}_{l}$ . Furthermore, each point r on the detector surface $\delta \Sigma^{\prime }$ has an acceptance cone with the apex at r , half-angle β , and axis unit vector $\hat{\text{u}}=-{\hat{\text{s}}}_{k}$ . An arbitrary point Y on the surface of this cone is defined by $(\text{Y}-\text{r})\cdot \hat{\text{u}}=|\text{Y}-\text{r}|\cos\beta$ . For convenience, we consider a double infinite cone, ${\left[(\text{Y}-\text{r})\cdot \hat{\text{u}}\right]}^{2}=(\text{Y}-\text{r})\cdot (\text{Y}-\text{r})\cos^{2}\beta$ . The intersection point(s) P between this cone and the incident ray satisfy the equations

(A1)
$$\text{P}={\text{r}}_{\text{s}}+\xi {\hat{\text{s}}}_{l},$$

(A2)
$${\left[(\text{P}-\text{r})\cdot \hat{\text{u}}\right]}^{2}-(\text{P}-\text{r})\cdot (\text{P}-\text{r})\cos^{2}\beta =0.$$

Substituting Eq. (A1) into Eq. (A2), we obtain

(A3)
$${\left[({\text{r}}^{\prime }+\xi {\hat{\text{s}}}_{l})\cdot \hat{\text{u}}\right]}^{2}-({\text{r}}^{\prime }+\xi {\hat{\text{s}}}_{l})\cdot ({\text{r}}^{\prime }+\xi {\hat{\text{s}}}_{l})\cos^{2}\beta =0\;,$$

where ${\text{r}}^{\prime }={\text{r}}_{\text{s}}-\text{r}$ . Further, Eq. (A3) can be written in the quadratic form $a\xi^{2}+b\xi +c=0$ , where $a={\left({\hat{\text{s}}}_{l}\cdot \hat{\text{u}}\right)}^{2}-\cos^{2}\beta$ , $b=2(({\hat{\text{s}}}_{l}\cdot \hat{\text{u}})({\text{r}}^{\prime }\cdot \hat{\text{u}})-{\hat{\text{s}}}_{l}\cdot {\text{r}}^{\prime }\cos^{2}\beta )$ , and $c={\left({\text{r}}^{\prime }\cdot \hat{\text{u}}\right)}^{2}-{\text{r}}^{\prime }\cdot {\text{r}}^{\prime }\cos^{2}\beta$ . Determining the points of intersection between the incident ray and the acceptance cone is equivalent to solving for ξ , subject to the conditions ξ>0 and $\xi {\text{s}}_{l,z}<L$ , which ensure that the intersection points are within the medium. Also, since we have considered a double infinite cone, the shadow cone is omitted by only allowing intersections for which $(\text{P}-\text{r})\cdot \hat{\text{u}}>0$ . It can be shown that, for small source-detector separation and positions on the detector far from the center, $b^{2}-4ac<0$ , and there are no real solutions. Δθ is calculated as the angle between the two vectors from the cone apex to each of the intersection points:

(A4)
$$\Delta \theta =\cos^{-1}\left[\frac{\left({\text{P}}_{1}-\text{r})\cdot ({\text{P}}_{2}-\text{r}\right)}{|{\text{P}}_{1}-\text{r}||{\text{P}}_{2}-\text{r}|}\right]\;,$$

where the intersection points ${\text{P}}_{1,2}={\text{r}}_{\text{s}}+\xi_{1,2}{\hat{\text{s}}}_{l}$ are determined using the solutions $\xi_{1,2}$ .
